# The microbiome of alpine snow algae shows a specific inter-kingdom connectivity and algae-bacteria interactions with supportive capacities

**DOI:** 10.1038/s41396-020-0677-4

**Published:** 2020-05-18

**Authors:** Lisa Krug, Armin Erlacher, Katharina Markut, Gabriele Berg, Tomislav Cernava

**Affiliations:** 1 Institute of Environmental Biotechnology, Graz University of Technology, Petersgasse 12, 8010 Graz, Austria; 2 ACIB GmbH, Petersgasse 14, 8010 Graz, Austria

**Keywords:** Microbial ecology, Microbial ecology

## Abstract

Mutualistic interactions within microbial assemblages provide a survival strategy under extreme conditions; however, little is known about the complexity of interaction networks in multipartite, free-living communities. In the present study, the interplay within algae-dominated microbial communities exposed to harsh environmental influences in the Austrian Alps was assessed in order to reveal the interconnectivity of eukaryotic and prokaryotic inhabitants. All analyzed snowfields harbored distinct microbial communities. Network analyses revealed that mutual exclusion prevailed among microalgae in the alpine environment, while bacteria were mainly positively embedded in the interaction networks. Especially members of *Proteobacteria*, with a high prevalence of *Oxalobacteraceae*, *Pseudomonadaceae*, and *Sphingomonadaceae* showed genus-specific co-occurrences with distinct microalgae. Co-cultivation experiments with algal and bacterial isolates confirmed beneficial interactions that were predicted based on the bioinformatic analyses; they resulted in up to 2.6-fold more biomass for the industrially relevant microalga *Chlorella vulgaris*, and up to 4.6-fold increase in biomass for the cryophilic *Chloromonas typhlos*. Our findings support the initial hypothesis that microbial communities exposed to adverse environmental conditions in alpine systems harbor inter-kingdom supportive capacities. The insights into mutualistic inter-kingdom interactions and the ecology of microalgae within complex microbial communities provide explanations for the prevalence and resilience of such assemblages in alpine environments.

## Introduction

Microalgae and bacteria can form complex, inter-kingdom microbial communities in various natural environments and exchange different metabolites for mutualistic support [1]. While freshwater and marine habitats are commonly analyzed to decipher microalgae–bacteria interactions [2–4], less is known about interactions in similar assemblages that are found on snowfields in alpine environments. These unique terrestrial habitats are highly vulnerable to climate change and will become scarcer in the future. Until recently, the biodiversity in cryophilic ecosystems was underestimated; novel molecular and bioinformatic tools facilitated the discovery of the unexpected complexity of microbial biodiversity and functionality present in glaciers, snow and ice fields [5]. Due to the important role as primary producers and the release of dissolved organic nutrients, microalgae allow heterotrophic microorganisms to co-exist in their surroundings. Such heterotrophic microorganisms include bacteria that not only decompose organic matter but that can also support higher eukaryotes in return by nutrient exchange and complex communication systems [6, 7]. Various metabolite exchanges including essential micro- and macronutrients were observed so far for algae-bacteria interactions in different habitats and in the context of coevolution and mutualistic support [8–10]. Several plant growth-promoting bacteria also harbor potential to stimulate algal growth by releasing essential minerals, vitamins, auxins, and quorum sensing signaling molecules [11–14].

Microalgae have attracted considerable interest worldwide in the last decades, mainly due to their applicability in the renewable energy, biopharmaceutical, and nutraceutical industry [15, 16]. However, industrial cultivation faces different challenges [17], and might be improved by implementing naturally inter-species communities in bioreactors. For example, symbiotic cultures of microalgae and bacteria provide a viable strategy for the elimination of unwanted, contaminating bacteria in aquaculture systems [18]; this is mainly due to the principle of competitive exclusion, also known as Gause’s law. The principle states that “complete competitors cannot co-exist” [19]; ecological niches occupied by beneficial microorganisms are less accessible for microorganisms detrimental for microalgae. By tracking down the sophisticated algae-bacteria interactions in natural environments, the design of beneficial, synthetic microbial communities becomes a more tangible tool for biotechnological applications.

In the present study, the focus was on the decipherment of microbial community structures on differently colored snowfields sampled at two geographically distant locations representative for the Central Alps in Austria; freshwater samples served as references to confirm expected differences in population structures between terrestrial (snowfields) and limnetic (freshwater from permanent water bodies) ecosystems, as the investigated environments show differing conditions in terms of nutrient availability, temperature, and light intensity. The main objective of the study was to characterize inter-kingdom interactions in seasonally occurring, nutrient-poor snowfields, where microorganisms have to adapt to low temperatures and high UV radiation and are therefore considered extreme environments. We hypothesized that mutualistic inter-kingdom relationships would support persistence of the occurring microorganisms under these adverse conditions. By applying co-occurrence network inferences, we focused on the identification of predominant correlations between microalgae and bacteria and the detection of putatively beneficial constituents within the microalgae-associated bacterial community. Bacterial isolates from the same sampling locations were screened for their potential to produce micro- and macronutrients attributable to increased biomass formation of microalgae (i.e., phytohormones and siderophores). In order to confirm positive interactions, bacterial isolates obtained from differently colored snowfields and freshwater samples were co-cultivated with the microalgae *Chlorella vulgaris* BRK1 to assess their direct impact on biomass formation of an industrially relevant microalgae model. Additional co-cultivation experiments with the cryophilic alga *Chloromonas typhlos* SAG 26.86 were performed to confirm the symbiotic character of algae–bacteria interactions on snowfields. The obtained results broaden our knowledge related to inter-kingdom interactions in extreme environments and provide a basis for upcoming biotechnological developments.

## Material and methods

### Sampling procedure

**Fig. 1:**
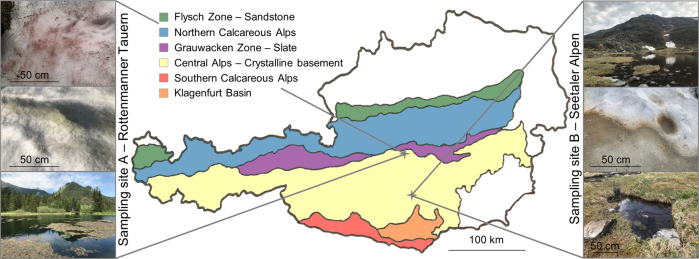
Locations of the sampling sites in the Austrian Alps. Red and green snowfields were sampled at site A (Rottemanner Tauern) while red and orange snowfields were sampled at site B (Seetaler Alpen). In addition, two freshwater samples in close proximity of the snowfields were sampled at each sampling site.

Snow and water samples were collected on May 30 and May 31, 2017 from two geographically distant locations in the Austrian Central Alps (sampling site A–Rottenmanner Tauern, N47° 26′ 24.695″ E14° 34′ 34.556″; sampling site B—Seetaler Alpen, N47° 3′ 56.128″ E14° 34′ 0.318″). The geographic locations of the sampling sites and examples of colored snowfields are shown in Fig. 1. Red snowfields were sampled on both sites while green and orange snow could only be found either at sampling site A or sampling site B, respectively. In addition, water samples of two separate permanent water bodies were obtained from the littoral zone from both sampling sites; the lakes were located within 3 km from the sampled snowfields. The sampled snowfields covered areas of up to 50 m^2^, where colored snow patches were locally distributed with areas of ~2 m^2^. Orange snow at site B was sampled at interfaces between snow and ice derived from frozen snow melt, while green and red snowfields were sampled at their surface. Samples were obtained in 3–5 biological replicates from each colored snow patch by scratching and collecting a total volume of 50 ml from the snow surface or the snow–ice interface in sterile 50-ml tubes (Sigma-Aldrich, Missouri, USA). Samples were stored on ice during transportation until further processing. Freshwater samples were obtained in triplicates by collecting 50 ml surface water in sterile 50-ml tubes. For further analyses all colored snow samples were melted and all samples thoroughly homogenized before further processing. Microalgae occurring in differently colored snowfields were visualized microscopically using a light microscope (Leitz; Wetzlar, Germany) at ×400 magnification (Fig. S1; Supplementary Material File 1).

### Isolation of microalgae-associated bacteria

In order to isolate microorganisms from snowfield and freshwater samples, dilution series were plated on nutrient agar (NA), Reasoner’s 2A agar (R2A), and modified Bolds Basal Medium agar plates (mBBM) containing 250 mg/l NaNO_3_, 175 mg/l KH_2_PO_4_, 75 mg/l K_2_HPO_4_, 75 mg/l MgSO_4_ × 7H_2_O, 25 mg/l CaCl_2_, 25 mg/l NaCl, 2.6 mg/l H_3_BO_3_, 5 mg/l FeSO_4_ × 7H_2_O, 8.8 mg/l ZnSO_4_ × 7H_2_O, 1.4 mg/l MnCl_2_ × 4H_2_O, 1.4 mg/l MoO_3_, 1.6 mg/l CuSO_4_ × 5H_2_O, 0.5 mg/l Co(NO_3_)_3_ × 6H_2_O, 0.5 mg/l EDTA, 0.3 mg/l KOH, 0.017 mg/l vitamin B_12_, 0.013 mg/l 4-aminobenzoate, 0.003 mg/l biotin, 0.013 mg/l nicotinic acid, 0.017 mg/l hemicalcium-pentathenate, 0.05 mg/l pyridoxamine-HCl, 0.033 mg/l thiaminiumdichlorid, 0.0091 mg/l thioctic-acid, 0.01 mg/l riboflavin, 0.0049 mg/l folic acid, and 18 g/l agar–agar. Vitamins and heat-sensible components were added after autoclaving by sterile filtration (0.20 µm pore size). Petri dishes with NA and R2A were incubated for 3 days at room temperature; those with mBBM-agar were incubated at 23 °C at a light dark cycle (L:16/D:8). The lighting was supplied by cool-white fluorescent lamps TL-D 36 W/840 REFLEX Eco (Philips, Amsterdam, Netherlands) with an intensity of 3350 lm for 3 days. A total number of 470 bacterial isolates were randomly selected from snowfield and freshwater samples and subcultured on the respective media.

### Identification of bacterial isolates and phylogenetic analysis of beneficial strains

Bacterial genomic DNA from pure cultures was extracted by mechanical disruption. Bacterial colonies from agar plates were resuspended in 300 µl sterile NaCl (0.85%) and transferred in sterile Eppendorf tubes filled with glass beads and subsequently processed in a FastPrep FP120 (MP Biomedicals, Heidelberg, Germany) instrument. After centrifugation at 3,000 rpm for 5 min the supernatant was transferred in sterile 1.5 ml tubes and served as template for the PCR. Subsequently, 16S rRNA gene fragments were amplified using primer 27F (5′-AGA GTT TGA TCM TGG CTC AG-3′) and 1492R (5′-CGG TTA CCT TGT TAC GAC TT-3′). The PCR was performed in a total reaction volume of 30 µl containing 16.20 µl ultrapure water, 6.00 µl Taq&Go [5×] (MP Biomedicals, Heidelberg, Germany), 1.50 µl of each primer [10 µM], 1.80 µl MgCl_2_ [25 mM], and 3 µl template (95 °C, 4 min; 30 cycles of 95 °C, 30 s; 57 °C, 30 s; 72 °C, 90 s; final extension at 72 °C, 5 min). The PCR products were purified using Wizard SV Gel and PCR-Clean-Up System (Promega Corporation, Madison, Wisconsin, USA) and DNA concentration was measured using a Nanodrop UV–Vis spectrophotometer (Thermo Fisher Scientific, Waltham, MA, USA), diluted accordingly and sent for Sanger sequencing (LGC Genomics GmbH, Berlin, Germany). Resulting 16S rRNA gene fragment sequences were then blasted against the NCBI nucleotide collection database excluding uncultured and environmental samples using the BLAST algorithm [20]. A phylogenetic tree that is based on 16S rRNA gene sequence alignments was constructed with MEGA X [21]. The evolutionary history was inferred by using the Neighbor-Joining method [22]. The percentage of replicate trees in which the associated taxa clustered together in the bootstrap test (1000 replicates) was separately assessed [23]. Evolutionary distances were computed using the Maximum Composite Likelihood method [24] and are provided as base substitutions per site.

### Microbiome analyses with bacterial and eukaryotic amplicon libraries

#### Total community DNA extraction and barcoding

For each sample 50 ml melted snow/freshwater was thoroughly homogenized and total community DNA was extracted from 2-ml aliquots of the respective sample using the FastDNA Kit for Soil (MP Biomedicals, Heidelberg, Germany) according to the manufacturer’s instructions. The 16S rRNA gene fragment sequences were amplified in three technical replicates covering the hypervariable region V4 using the Unibac II 515 f (5′-GTG YCA GCM GCC GCG GTA A-3′) and 806r (5′-GGA CTA CHV GGG TWT CTA AT-3′) primer pair [25], which included sample-specific barcodes and Illumina-sequencing adapters. Peptide nucleic acid was added to the PCR mix to prevent the amplification of mitochondrial (mPNA) and plastidial (pPNA) DNA from eukaryotes [26]. The PCR was performed by using a total volume of 30 μl containing 20.15 μl ultrapure water, 6 μl Taq&Go [5×], 1.2 μl of each primer (5 μM), 0.225 μl pPNA [100 μM], 0.225 μl mPNA [100 μM], and 1 μl DNA template. The cycling program was adjusted to an initial denaturation temperature at 96 °C for 5 min, followed by 30 cycles of 96 °C for 1 min, 78 °C for 5 s, 54 °C for 1 min, and 74 °C for 1 min. The final extension was done at 74 °C for 10 min. PCR products of respective samples were quality checked by gel electrophoresis. Quality (amount, concentration) of the PCR products from freshwater samples was found to be insufficient during the quality check; therefore, another PCR was performed with primer pair 27F and 1492R prior to the described PCR reaction in a nested approach. The PCR products were then purified using the Wizard SV Gel and PCR-Clean-Up System according to the manufacturer’s protocol and served than as template for further amplifications.

From the same total community DNA extracts, 18S rRNA gene fragments of the eukaryotic community were amplified in a first PCR with the primer pair 1391f (5′-GTA CAC ACC GCC CGT C-3′) and EukBr (5′-TGA TCC TTC TGC AGG TTC ACC TAC-3′) targeting the variable region 9 (V9) of the 18S rRNA gene sequence [27]. Each forward and reverse primer contained a specific primer pad (TATGGTAATT/AGTCAGCCAG) and linker (GT/GG), as described in the protocols and standards section of the Earth Microbiome Project (www.earthmicrobiome.org) [28]. PCR reactions (20 µl) were conducted in triplicates and contained 14.6 µl ultrapure water, 4 µl Taq&Go [5×], 0.2 µl of forward and reverse primer each [10 µM] and 1 µl extracted DNA template. The cycling program was adjusted to the following settings: 98 °C, 5 min; 10 cycles of 98 °C, 10 s; 53 °C, 10 s; 72 °C, 30 s; 20 cycles of 98 °C, 10 s; 48 °C, 30 s; 72 °C, 30 s; final extension 72 °C, 10 min. PCR products of respective samples were quality checked by gel electrophoresis. The quality (amount, concentration) of the PCR products of freshwater samples was found to be insufficient; therefore, another PCR was performed prior to the described PCR reaction for nested approaches with primer pair NS1 (5′-GTA GTC ARA RGC CTT GTC TC-3′) and NS8 (5′-TCC GCA GGT TCA CCT ACG GA-3′) in a reaction mix (30 µl) containing 16.2 µl ultrapure H_2_O, 6 µL Taq&Go [5×], 1.2 µl of each primer [10 µM], 2.4 µL MgCl_2_ [25 mM] and 3 µl DNA template. PCR products were then purified using the Wizard SV Gel and PCR-Clean-Up System according to the manufacturer’s protocol and served as template for further amplifications. For multiplexing, sample-specific Golay barcodes were attached to the primer pad on forward and reverse primer, respectively. Barcoded sequences were pooled and purified according to the Wizard SV Gel and PCR-Clean-Up System. Equimolar DNA concentrations of each barcoded amplicon (bacterial and eukaryotic) were then sent for paired-end Illumina HiSeq sequencing (read length: 2 × 300 bp) to GATC Biotech AG (Konstanz, Germany).

#### Initial bioinformatic analyses of 16S rRNA and 18S rRNA gene amplicons

Raw sequencing data preparation, including joining forward and reverse read pairs was done using the software package QIIME 1.9.1. After removing barcodes, primer and adapter sequences reads as well as metadata were imported into QIIME 2 (2018.11 release). Further analyses of sequencing data were performed using the QIIME 2 pipeline according to tutorials provided by the QIIME developers [28]. The DADA2 algorithm [29] was used to demultiplex and denoise truncated reads. Chimeras were identified by using the VSEARCH ‘uchime_denovo’ method [30] and subsequently removed. Respective reads were then summarized in a feature table. The 16S rRNA dataset was normalized to 98,216 reads per sample to account a variation in the samples reaching from 719,183 to 98,216 reads. Features were then collapsed on genus level and a reduced table containing only highly abundant taxa (>0.1% mean relative abundance) was used for generating bar charts. In analogy, the 18S rRNA dataset was normalized to 21,347 reads per sample to account for the variation in the samples reaching from 788,817 to 21,347 reads. Rarefied feature tables served as input for alpha and beta diversity analyses and statistics using QIIME 2 core diversity metrics. Principal coordinate analysis (PCoA) plots were constructed by calculating the unweighted UniFrac distance matrix [31]. Phylogenetic metrics were constructed by aligning representative sequences using the mafft program [32]. After the multiple sequence alignment was masked and filtered a phylogenetic tree was generated with FastTree [33]. The taxonomic analysis was based on a customized naive-Bayes classifier trained on 16S and 18S rRNA gene OTUs clustered at 99% similarities with the SILVA128 database release and trimmed to a length of 400 bp (16S) and 200 bp (18S) respectively. Statistics were calculated within QIIME 2 using analysis of similarity (ANOSIM) in order to determine statistically significant differences in the compositions of different microbial communities. The datasets used and/or analyzed during the current study are available in the ENA repository (https://www.ebi.ac.uk/ena) under the accession number PRJEB31713.

#### Complementary network inference analyses with combined datasets

In order to assess associations between all features (sequence variants) within the microalgal and proteobacterial fraction across different sample types, an association network inference analysis was implemented. Co-occurrence network analyses were calculated and rendered using Cytoscape version 3.7.0 [34] and the CoNet add-on [35]. In the CoNet add-on, Pearson and Spearman correlation measurements, Bray–Curtis and Kullback–Leibler dissimilarity matrices and the mutual information option, were used to ensemble inferences. *P* values are calculated by the software from permutation and bootstrap distributions (1000 iterations) including Benjamini–Hochberg multiple test correction. Highly significant inferences (*p* = 0.0004, *q* = 0.0004) were retained. As an input, reduced, rarefied feature tables containing only features assigned to *Archaeplastida* and *Proteobacteria* with a mean relative abundance of at least 0.1% on snowfields were used for inference analyses. Taxonomic assignment of features represented in the network was conducted using the blast algorithm against the NCBI nt collection.

#### Detection of extended correlations patterns between microalgae and their associated microbiota

In order to provide a larger overview of correlations between selected eukaryotic groups and bacteria in snowfields, correlation coefficient measures were employed with features collapsed at genus level as input within METAGENassist [36]. The software package was used to calculate co-occurrence patterns within the eukaryotic and bacterial communities on snowfields based on Kendall’s tau rank correlation and visualize them with the integrated heatmap tool. Following the normalization of the datasets according to their sequencing depth, features assigned to the phyla *Chlorophyceae*, *Chrysophyceae,* and *Basidiomycota* were extracted from the 18S rRNA dataset and manually blasted against the NCBI nucleotide collection database. For calculating correlations between microalgae and bacteria, the bacterial feature table was reduced by retaining only features with a mean relative abundance of at least 0.1% on snowfields. Features with the same taxonomic assignment were combined and unassigned reads were excluded from the dataset.

### Physiological characterization of cultured bacterial isolates and algal biomass quantification

All bacterial isolates were screened for the production of N-acylhomoserine lactone (AHL; 37–39), indole-3-acetic acid production [40] and the production of siderophores. Microalgal biomass was assessed with a fluorometric approach by correlating algal cell numbers to the chlorophyll A content. The methods are described in more detail in the Supplementary Material and Methods section (Supplementary Material File 2).

### Co-cultivation experiments to evaluate the potential of bacteria to promote microalgae growth

The growth-promotion assay was performed with the unicellular microalga strain *C. vulgaris* BRK1, isolated from a photobioreactor [41] and *C. typhlos* SAG 28.86—a cryophilic alga isolated from snow (SAG Strain No. 28.86; Sammlung von Algenkulturen Göttingen; Göttingen, Germany) by plating respective samples on mBBM-agar and subsequent subculturing [41]. For initial cultivation, 50 ml mBBM were inoculated with a single colony of *C. vulgaris* and grown for 1 day at 23 °C at a light dark cycle L:16/D:8. The *C. typhlos* preculture was obtained by inoculating 50 ml mBBM and subsequent culturing for 4 days at 4 °C at a light dark cycle L:16/D:8. The microalgae were cultivated until the axenic cultures reached a cell density of 2.75 ± 0.60 × 10^6^ for *C. vulgaris* and 5.4 ± 0.9 10^5^ for *C. typhlos*. For the prescreening, growth-promoting experiments were performed with *C. vulgaris* in eight replicates with all 470 bacterial isolated from snowfield and freshwater samples. The growth-promotion assay was repeated in 18-fold replication with bacterial isolates indicating growth-promotion during the prescreening in order to facilitate statistical analyses. Only those bacterial strains that showed positive effect on the growth of *C. vulgaris* were further tested for their effect on *C. tyhphlos*. For co-cultivation experiments, axenic algal cultures were transferred in 96-well plates, each well containing 200 µl algae culture. The growth-promotion assay was not adjusted to a specific bacterial density, thus microalgae cultures were inoculated with bacteria using sterile toothpicks. The significance of the results was tested using the IBM SPSS program (version 23.0; IBM Corporation, NYC, NY, USA). All data were analyzed using the Student’s paired *t* test at *p* < 0.01.

## Results

### Bacterial and eukaryotic community structures were highly habitat-specific

**Fig. 2:**
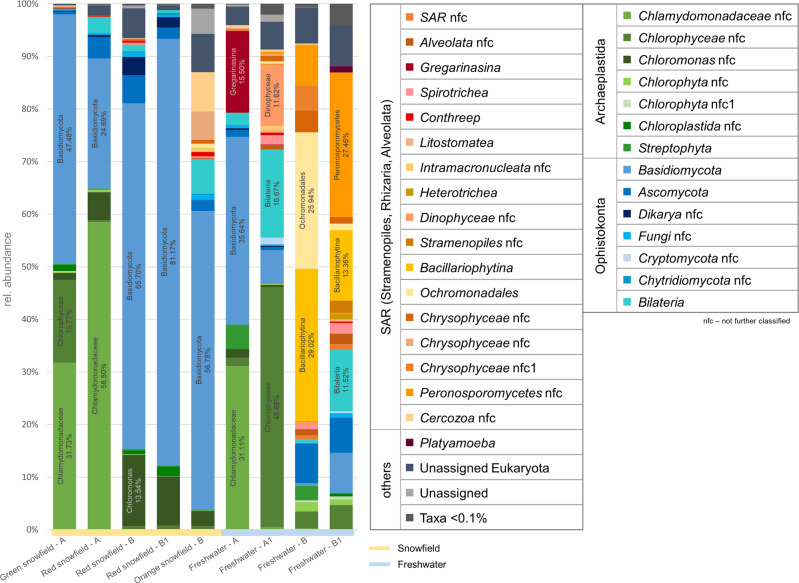
Taxonomic classification of the eukaryotic community inhabiting differently colored snowfields and freshwater sampled at two geographically distant locations in the Austrian Alps. Bar charts represent the prevailing fraction of the eukaryotic community with a relative occurrence ≥0.1% in the whole dataset. Sampling site A: Rottenmanner Tauern; sampling site B: Seetaler Alpen. Labels and the relative abundances of highly abundant taxa (>10% rel. abundance) are included in the respective bar chart.

**Fig. 3:**
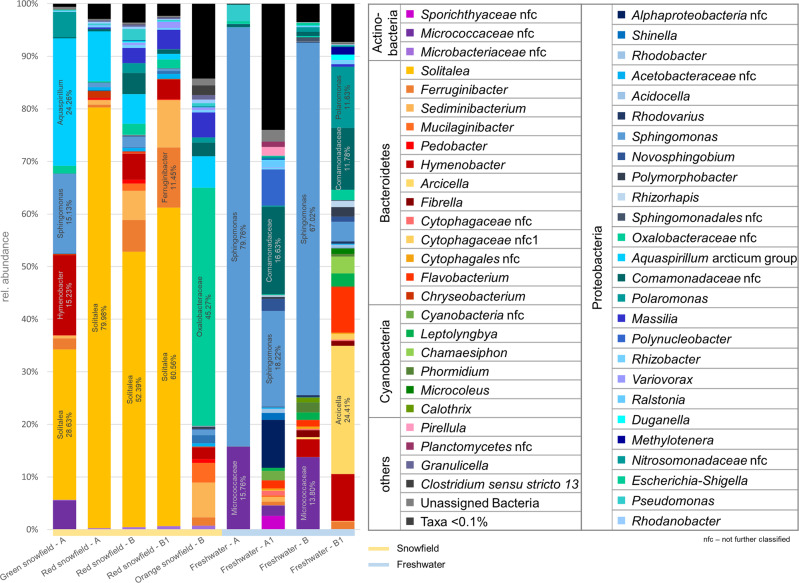
Composition of the bacterial community on differently colored snowfields and in freshwater sampled at two geographically distant locations in the Austrian Alps. Taxonomic assignments were conducted at genus level. Bar charts represent the fraction of abundant taxa in the bacterial community with a relative abundance ≥0.1% in the whole dataset. Sampling site A: Rottenmanner Tauern: sampling site B: Seetaler Alpen. Labels and the relative abundances of highly abundant taxa (>10% rel. abundance) are included in the respective bar chart.

**Fig. 4:**
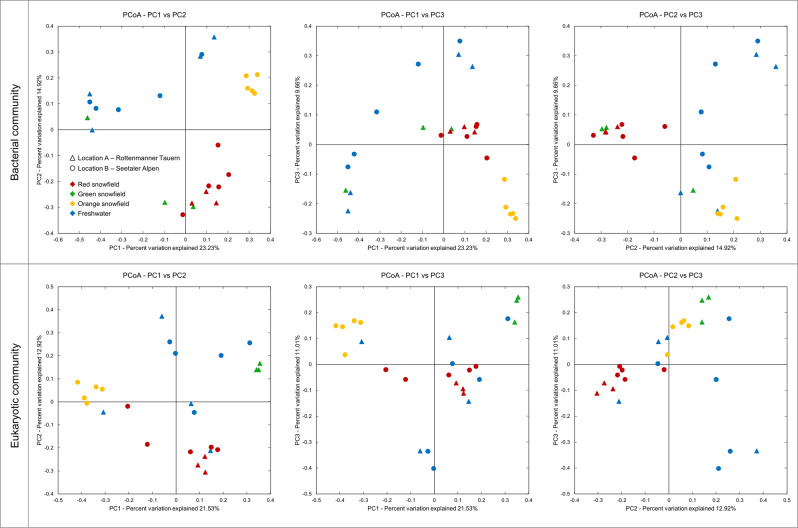
PCoA plots of bacterial and eukaryotic communities on differently colored snowfields and freshwater sampled at two geographically distant locations in the Central Alps. The community clustering is based on Bray–Curtis dissimilarities (unweighted UniFrac). Different sampling locations and sample types are indicated with distinct shapes and colors, respectively.

*Chlamydomonas nivalis* was the predominant algal species within the eukaryotic communities (Table S1; Supplementary Material File 1) and *Solitalea koreensis* in the prokaryotic communities (Table S2; Supplementary Material File 1). The overall bacterial and eukaryotic community compositions (Figs. 2 and 3) are detailed in Supplementary Results section (Supplementary Material File 2). Bacterial community compositions substantially differed when differently colored snowfields in close proximity were compared. In contrast, when snowfields of the same color but from geographically distant locations were analyzed, compositional commonalities in inter-kingdom communities indicated possible relationships between co-occurring microalgae/eukaryotes and bacteria. Statistical analyses based on ANOSIM tests were performed in order to identify factors that had the highest effect on the composition of bacterial communities. We have excluded that the sampling location has a significant impact on bacterial communities (Fig. 4), suggesting that their composition is affected by other factors. The habitat type (snowfield and freshwater) had the pronounced effect on the present bacteria, highly significant differences between terrestrial and limnetic communities were evident (*R* = 0.594; *p* = 0.001). Congruent observations were found for the eukaryotic communities, the habitat type had a significant influence on the structure of the eukaryotic fraction of the microbiome (*R* = 0.223; *p* = 0.008; Tables S3 and S4; Supplementary Material File 1).

### Snowfield color reflects the bacterial and eukaryotic community structure

When the similarities of differently colored snowfields obtained from geographically distant locations were compared, we observed a high correlation between the snow color and the composition of the bacterial community (*R* = 0.841; *p* = 0.001). Comparisons of all red colored snowfields indicated no significant differences in the composition of bacterial members of the microbiota (*R* = −0.015; *p* = 0.501). The same observation was made when the eukaryotic community composition was analyzed on differently colored snowfields. Again, the color of snowfields independent from sampling site had the highest and most significant impact (*R* = 0.903; *p* = 0.001), while red colored snowfields showed no differences between the geographically separated locations (*R* = 0.067; *p* = 0.229). For limnetic systems, the sampling site had only a minor effect on the microbiome composition (18S: *R* = 0.075, *p* = 0.265; 16S: *R* = 0.194, *p* = 0.116; Tables S3 and S4; Supplementary Material File 1). Complementary alpha diversity analyses indicate a sufficient sampling depth for both bacterial and eukaryotic datasets allowing robust comparatistics between samples (Fig. S2; Supplementary Material File 1).

### Co-occurrence network analyses revealed mutual-exclusion patterns between microalgae

**Fig. 5:**
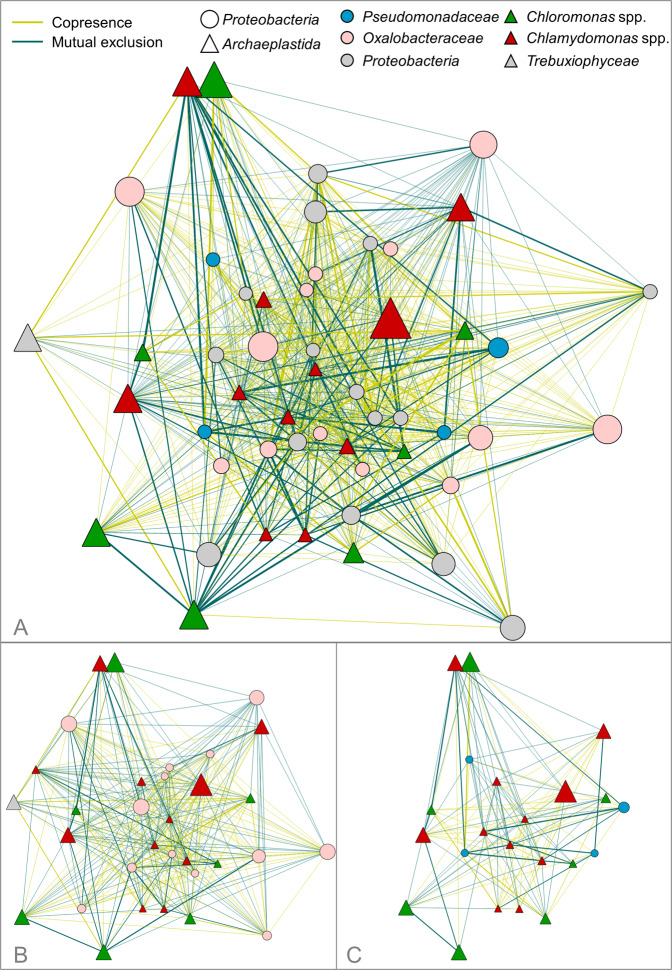
Co-occurrence network analyses of bacterial and eukaryotic taxa found on differently colored snowfields. Significant (*p* < 0.0004; *q* < 0.0004) co-occurrences (blue edges) and mutual-exclusion patterns (yellow edges) between features assigned to *Proteobacteria* and *Archaeplastida* (**a**) are displayed. The node size corresponds to the relative abundance of respective taxa. The edge width indicates the significance of interactions. The co-occurrence network was reduced in order to highlight interactions between algae and *Oxalobacteraceae* (**b**) and *Pseudomonadaceae* (**c**).

In order to elucidate specific co-occurrences between *Proteobacteria* and *Chlorophyta* on snowfields, association network inference analyses were conducted. The resulting network comprised 51 nodes with a network density of 0.655 (Fig. 5). The algal community was represented by 12 features assigned to *Chlamydomonas*, six assigned to *Chloromonas* and one feature assigned to *Scotiella*. In the proteobacterial fraction, the prevalent representatives were members of the *Acetobacteriaceae* family (four features), *Comamonadaceae* (four features), *Oxalobacteraceae* (13 features), *Pseudomonadaceae* (four features), and *Sphingomonadaceae* (three features). The network inference analyses revealed that *Massilia*, a member of the *Oxalobacteraceae* family, had predominantly positive interconnections (31 of a total number of 39 links). Moreover, prevalently positive interactions were observed between *Massilia* and features assigned to *Chloromonas*, while exclusively negative interactions were observed between *Massilia* and features assigned to *Chlamydomonas*. Additional features assigned to taxa belonging to the *Oxalobacteraceae* family showed similar correlations (Fig. 5b). Analogous observations were obtained when the interconnections of *Pseudomonadaceae* and algal features were assessed; negative interconnections prevailed between *Pseudomonas* and *Chlamydomonas* while positive interconnections predominate with *Chloromonas* (Fig. 5c). Remarkably, *Sphingomonas* was amongst the features with the most interconnections. Out of a total number of 38 interconnections of a distinct feature, 30 indicated a co-occurrence with microalgae; seven out of eight edges indicated mutual exclusion between *Sphingomonas* and features assigned to *Chlamydomonas*, while exclusively positive correlations are found between *Sphingomonas* and features assigned to *Chloromonas*. Moreover, except one negative correlation with a feature assigned to *Aquaspirillum*, all other interconnections of *Sphingomonas* with bacterial taxa indicated co-occurrences on snowfields. Within the algal community on snowfields, features assigned to *Chloromonas* showed the most positive interconnections (in total 143 links). Negative interconnections were frequently detected between a feature assigned to *Aquaspirillum* (47 links) and several bacterial features including all members of the *Oxalobacteraceae* family; mutual-exclusion patterns were also observed between *Aquaspirillum* and five features assigned to *Chloromonas*. When holistically assessed, co-occurrence patterns prevailed within the bacterial kingdom. On the contrary, mutual exclusions prevailed within the investigated members of the *Chlorophyta* phylum and features assigned to different genera.

### Correlation analyses suggested specific inter-kingdom co-occurrences

**Fig. 6:**
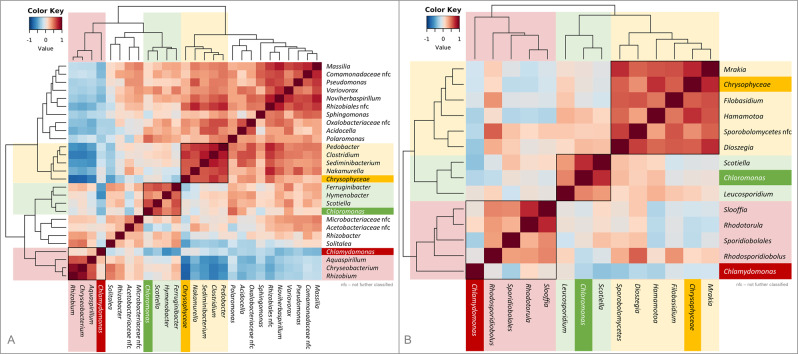
Correlation patterns between microalgae and their associated bacteria and fungi on differently colored snowfields. Distinct correlations of microalgae and their associated bacteria (**a**) and basidiomycetous yeasts (**b**) were observed for identified features summarized at genus level. Correlations were calculated for microalgal (*Chlorophyceae, Chrysophyceae*) and bacterial features with an abundance ≥0.1% in the snowfield samples (**a**). To identify correlations between algae and basidiomycetous yeasts, features assigned to *Basidiomycota* were manually blasted against the NCBI nucleotide collection. Features with the same taxonomic assignment at genus level were combined. The analyses are based on the Kendall’s tau rank correlation.

Extended correlation analyses between abundances of bacterial and algal genera on snowfields indicated inter-kingdom connections between distinct inhabitants of the cryophilic environments (Fig. 6a); *Chrysophyceae* showed strong positive correlations with *Pedobacter, Clostridium, Sediminibacterium*, and *Nakamurella*, while negative correlations were observed with *Aquaspirillum, Chryseobacterium*, and *Rhizobium*. On the contrary, those taxa (*Aquaspirillum, Chryseobacterium* and *Rhizobium*) correlated positively with *Chlamydomonas*. For *Chloromonas* strong correlations were identified with the bacterial genera *Ferruginibacter* and *Hymenobacter*. Correlation analyses of occurrences of microalgae (*Chlorophyta*, *Chrysophyta*) and basidiomycetous yeasts found on snowfields revealed similar results in terms of genus specificity (Fig. 6b). The strongest positive correlations were found between *Chrysophyceae* and several yeasts including the genera *Mrakia, Filobasidium, Hamamotoa, Sporobolomycetes*, and *Dioszegia*. *Chloromonas* showed strong positive correlations with *Leucosporidium*, while *Chlamydomonas* spp. correlated positively with *Rhodotorula*.

### Physiological characterization of bacteria isolated from snowfields and freshwater

The cultivable fraction of the bacterial community isolated from snowfields and freshwater ponds was screened for its potential to produce siderophores and auxins. In total 470 bacterial isolates were tested for their physiological properties attributable to microalgae growth promotion. A total of 52% tested isolates produced more than 3 µg/ml quantifiable auxins, whereas 12% excreted more than 15 µg/ml auxins into the cultivation media. When assessing the potential of bacterial isolates to produce siderophores, 34% of all tested isolates showed positive results. In a complementary co-cultivation approach with bacterial isolates, nine strains were identified, which significantly increased the biomass formation of *C. vulgaris* BRK1. The observed growth promotion ranged from 1.5- to 2.6-fold increased biomass formation with the employed model algae (Table S5; Supplementary Material File 1). Additional co-cultivation experiments with *C. typhlos* SAG 26.89, a cryophilic alga isolated from snow, revealed increased biomass formation of up to 4.6-fold when co-cultured with certain bacterial strains compared to axenic cultures. However, growth-promoting effects showed species specificity, as *Pseudomonas* sp. 1Ab3 led to growth inhibition of *C. typhlos*, while other *Pseudomonas* strains showed contrary effects. For three strains, which showed positive effects on the growth of *C. vulgaris*, no significant difference in algal cell count was observed with *C. typhlos* (Table S5, Supplementary Material File 1). Six of the promising strains were isolated from snowfields while three derive from freshwater samples. All isolates were identified as *Proteobacteria* by Sanger sequencing of 16S rRNA gene fragments; seven were identified as *Pseudomonas* spp. (five originated from red snowfields and two from freshwater samples). The remaining two isolates were assigned to *Aeromonas salmonicida*. (*Gammaproteobacteria*, *Aeromonadaceae*) isolated from freshwater and *Janthinobacterium* sp.—a member of the family of *Oxalobacteraceae* (*Betaproteobacteria*) isolated from an orange snowfield. Further characterization of growth-promoting strains revealed that two isolates (*A. salmonicidan* and *P. trivialis*, both isolated from freshwater taken at sampling site A) possess the ability to produce AHL. The evolutionary history of all growth-promoting isolates is displayed in Fig. S3 (Supplementary Material File 1). Table S5 (Supplementary Material File 1) includes the detailed results of the physiological characterization of the most promising algae growth-promoting bacterial isolates. The 16S rRNA gene fragment amplicon library was screened for occurrence of sequences of the growth-promoting strains; their relative abundances within the samples are included in Table S6 (Supplementary Material File 1).

## Discussion

To date, very few studies exist targeting microalgae-related microbiomes in their natural environment. We have selected an extreme environment exposed to elevated UV radiation, low temperatures and limited nutrients to study so far unexplored interactions between indigenous microalgae and prevalent bacterial communities. By investigating the bacterial and eukaryotic community structure on differently colored snowfields and in freshwater samples, a habitat-specific and location-independent bacterial and eukaryotic community composition was revealed. The inter-kingdom, co-occurrence network analyses indicated a prevalence of negative co-occurrence between microalgae on snowfields; microalgae most likely exclude each other by competing for nutrients and space following the competitive exclusion principle [19]. Detailed co-occurrence analyses of *Archaeplastida* and members of the bacterial families *Pseudomonadaceae* and *Oxalobacteraceae* indicated genus-specific algae-bacteria co-occurrences; similar genotype-specific interactions were previously described between higher plants and their associated microbiota [42, 43]. The color of the snowfields had the highest influence on microbial inhabitants, where the correlation was stronger for the eukaryotic community composition. Moreover, the sampling site had a minor and not significant effect on both, the bacterial and eukaryotic community composition on snowfields as well as in freshwater. This finding concurs with previous long-distance assessments, where community analyses of red colored snow in the Arctic and Antarctic indicated that bipolar taxa account for more than 37% of all reads, indicating the location-independent colonization of algae on snow [44]. Our results lead to the extended hypothesis that the structure of the microbial communities on snowfields might be partially driven by the occurrence of certain microalgal species.

*Chlamydomonas* and *Chloromonas* were predominant microalgal genera in red and green snowfields respectively, both are typical colonizers of snow [45–47]. Interestingly, green colored snowfields inhabited by *C. nivalis* are rarely found, because the green, flagellated cells have a very short reproductive phase before they form spores to better resist excessive irradiation, desiccation, low nutrient concentrations, and freeze-thaw cycles [48–50]. Previous studies have indicated that green and red snowfields might be successive stages [51]. However, in our study, the green color derived likely from *Chloromonas* spp. which are frequently detected on green snowfields; therefore, we assume that green and red snowfields are not always successive stages, but also can reflect independent phenomena. Results of cultivation-independent analyses are strongly supported by microscopic observations; distinct algal cell morphologies were observed for differently colored snow. *Spumella-*like flagellates (*Chrysophyceae*), predominant on orange snowfields, were identified among other members of the *Chrysophyceae* class in previous studies. The coloration was found to be due to pigments involved in the xanthophyll cycle, which is used by chrysophytes snow algae to cope with excessive light energy [52, 53].

Explorations of the bacterial community structure on differently colored snowfields revealed *Solitalea koreensi* (*Sphingobacteriaceae*) and other common inhabitants of polar and alpine environments. They are typical indicators for a highly specialized community in psychrophilic environments [46, 54–56]. The two prevalent bacterial phyla *Proteobacteria* and *Bacteroidetes* share the ability to rapidly degrade organic matter which may underpin a potential direct transfer of organic carbon from algae to bacteria [57]. Our results are in accordance with findings from previous studies investigating the bacterial and eukaryotic community composition on snowfields in the Pacific Northwest and in Japan [46, 56]. So far, highly similar community structures were identified at globally dispersed sampling sites, thus we assume highly conserved, evolutionary evolved, mutualistic relationships between certain microalgae and bacteria. We have implemented freshwater samples from both sampling sites in order to highlight the specificity of snowfield inhabitants.

In the eukaryotic communities, also members of the fungal phylum *Basidiomycota* prevailed on distinct snowfields. The most prominent features (*Rhodotorula*, *Leucosporidium*, and *Mrakia*) were previously reported as prominent inhabitants of cryospheres [58–60]. They are specialized to persist under extreme environmental conditions due to changes in the composition of membrane lipids, production of different cryoprotectants, and additional metabolic adaptions [61]. Our results are in accordance with previous findings, where species of the genus *Rhodotorula* have been described as enriched co-inhabitants of algae in red colored snow in the United States [58], while *Leucosporidium* and *Chloromonas* co-occurred on snow in the Canadian High Arctic [54]. Several species of the highly abundant genera *Rhodotorula, Leucosporidium*, and *Rhodosporidium* are able to produce rhodotorulic acid, a highly efficient iron chelating agent [62, 63] and have traits attributable to plant growth promotion [64–66] and thus may support algal communities in a mutualistic relationship. Correlation analyses between abundances of microalgae (*Chlorophyta* and *Chrysophyta*) and basidiomycetous yeasts on differently colored snowfields indicated that the presence of certain microalgae affects the co-occurring fungal community on snow.

In order to search for potentially beneficial algae-bacteria interactions [67], complementary growth-promotion experiments were conducted with the industrially relevant microalga *C. vulgais*. Within the obtained culture collection, 2% of the isolated bacteria were shown to significantly improve the growth of the implemented model organism; the majority was assigned to *Pseudomonas* spp. In a related study, Fu et al. investigated the interactions between a *Pseudomonas* sp. isolate and the microalga *Ulva prolifera* and found distinct bacterial genomic traits contributing to their symbiotic relationship [68]. In contrast, members of the bacterial family *Pseudomonadaceae* have also been reported to be able to produce algicidal compounds, indicating the specificity of growth-promoting and detrimental effects on different algae [69, 70]. These findings are also reflected by the implemented co-occurrence network analyses. Further support of the interaction specificity was provided by additional results of the cultivation-dependent analyses, where co-cultivation of only four out of the seven *Pseudomonas* spp. also led to significantly increased biomass formation in the cryophilic model algae *C. typhlos*. The influence of bacterial cell density was not assessed in the present study; however, we would expect that this factor influences the extend of the observed interactions as previously shown [71]. When physiological traits were analyzed, most of the growth-promoting *Pseudomonas* spp. were shown to produce siderophores and auxin. In seawater, microalgae often rely on iron from bacterial chelates [72], this might also facilitate interactions on snowfields. Amin et al. demonstrated a “carbon for iron mutualism” as dinoflagellate algae were involved in assimilation of iron complexed in siderophores and in return released dissolved organic matter in their surrounding and thereby support bacterial growth [73–75]. In the past, several studies focused on the effects of phytohormones on the performance and fitness of microalgae whereby improved biomass formation and lipid production were observed [76–80]. Overall, the present results provide evidence for evolutionary evolved relationships with inter-kingdom supportive capacities in alpine systems. Moreover, our results indicate that also fungal communities might be involved in the complex interplay; however, this remains to be confirmed with targeted experiments.

In natural habitats, algae–bacteria interactions are complex and essential for both partners. By investigating evolutionary evolved microbiome–microalgae associations in an extreme environment, we aimed to deepen the knowledge of specific algae–bacteria interactions. We found, that mutualistic associations involving members of three different kingdoms are (1) highly prevalent in the investigated alpine system, (2) directed in terms of the specificity of the interacting partners, and (3) that positive algae-bacteria interactions are highly efficient in increasing microalgal biomass without additional nutrient supply. Furthermore, we have identified different members of *Proteobacteria*, especially *Pseudomonas* spp. as cultivable constituents of the microbiome that can be employed to increase algal growth in artificial systems. These findings can be further exploited to increase yields and to provide stability together with reproducibility in industrial microalgae cultivation systems by targeted co-inoculations with compatible bacterial strains.

## Supplementary information

Supplementary Material File 1

Supplementary Material File 2
