# Supplementary material for: The microbiome of alpine snow algae shows a specific inter-kingdom connectivity and algae-bacteria interactions with supportive capacities
Source: ISME J. 2020 May 18;14(9):2197–210. doi: 10.1038/s41396-020-0677-4 (PMC7608445; doi:10.1038/s41396-020-0677-4)
Supplement: Supplementary file 1 — Supplementary Material File 1 [file 41396_2020_677_MOESM1_ESM.docx]

**Supplementary Material File 1**

**The microbiome of alpine snow algae shows a specific inter-kingdom connectivity and algae-bacteria interactions with supportive capacities**

**Lisa Krug**^1^**^,2^, Armin Erlacher^1^, Katharina Markut^1^, Gabriele Berg^1^ and Tomislav Cernava^1*^**

*^1^Institute of Environmental Biotechnology, Graz University of Technology, Petersgasse 12, 8010 Graz, Austria*

*^2^ACIB GmbH, Petersgasse 14, 8010 Graz, Austria*

**Correspondence:*

*Tomislav Cernava, Institute of Environmental Biotechnology,*

*Graz University of Technology, Petersgasse 12,*

*8010 Graz, Austria.*

*e-mail:* [*tomislav.cernava@tugraz.at*](mailto:gabriele.berg@tugraz.at)

**Running title:** Interactions within alpine microbial communities

**Submitted to:** ISME Journal

**Subject Category:** Microbial ecology and functional diversity of natural habitats

**Keywords:** microalgae; microbiome; snowfield communities; freshwater microorganisms; microbial interplay

**Table S1. Taxonomic assignment of highly abundant features within the 18S rRNA gene amplicon dataset.** The mean relative abundance [%] for the respective group of samples is shown, including closest taxonomic assignment when blasting sequences against the NCBI nucleotide collection database. Darker colors indicate higher abundances of respective feature.

| **Feature ID** | **Green snowfield** | **Red snowfield** | **Orange snowfield** | **Freshwater** | **Species** | **Family** | **Order** | **Class** | **Phylum** |
| --- | --- | --- | --- | --- | --- | --- | --- | --- | --- |
| c0ff0505b4d15b7e5ade5e1bca8e04b2 | 10.39 | 16.42 | <0.00 | 5.85 | *Chlamydomonas nivalis* | *Chlamydomonadaceae* | *Chlamydomonadales* | *Chlorophyceae* | *Chlorophyta* |
| 448c7b32f300024bc31e4b505d26605d | 3.39 | 18.31 | 5.94 | 1.75 | *Rhodotorula glutinis* | *Sporidiobolaceae* | *Sporidiobolales* | *Microbotryomycetes* | *Basidiomycota* |
| dd38b973c46378b067c9e51a51feb7be | 2.54 | 11.59 | 2.05 | 3.96 | *Slooffia cresolica* | *Sporidiobolaceae* | *Sporidiobolales* | *Microbotryomycetes* | *Basidiomycota* |
| 818f5dd22a6eb73b51bb811eabd35b63 | 0.01 | 0.01 | 0.00 | 9.12 | *Chlamydomonas angulosa* | *Chlamydomonadaceae* | *Chlamydomonadales* | *Chlorophyceae* | *Chlorophyta* |
| 44f43de574acf89e75ee02d1329e226c | 2.87 | 10.56 | <0.00 | 0.14 | *Leucosporidium antarcticum* | *Leucosporidiaceae* | *Leucosporidiales* | *Microbotryomycetes* | *Basidiomycota* |
| 920e8ad1272bf3b4e234533b72bed8f7 | 16.02 | 1.33 | 5.13 | 0.30 | *Leucosporidium antarcticum* | *Leucosporidiaceae* | *Leucosporidiales* | *Microbotryomycetes* | *Basidiomycota* |
| b06392de347d1cd0f4b357b012644e93 | <0.00 | 0.12 | 20.38 | 0.72 | *Mrakia frigida* | *Mrakiaceae* | *Cystofilobasidiales* | *Tremellomycetes* | *Basidiomycota* |
| 15ff9eb7803479bbb1c6bec335aefa7e | 0.00 | 0.00 | 0.00 | 5.87 | *Synedra berolinensis* | *Fragilariaceae* | *Fragilariales* | *Bacillariophyceae* | *Bacillariophyta* |
| 3002a1b9d3a1f003165347ea9c6f08a0 | 0.00 | <0.00 | 0.07 | 5.74 | *Spumella* sp. | *Chromulinaceae* | *Chromulinales* | *Chrysophyceae* | *Stramenopiles* |
| f66edbf1c5a94e9041ab838892e3b790 | 0.51 | 6.65 | 0.21 | 0.11 | *Chloromonas* sp*.* | *Chlamydomonadaceae* | *Chlamydomonadales* | *Chlorophyceae* | *Chlorophyta* |
| 1a9d461418f16b421c86dffcbc9888aa | 5.14 | 3.88 | 2.19 | 0.43 | *Rhodotorula glutinis* | *Sporidiobolaceae* | *Sporidiobolales* | *Microbotryomycetes* | *Basidiomycota* |
| 029a9185e535a94f2300b07860205bd5 | 15.52 | 0.13 | 0.05 | 0.08 | *Chloromonas actinochloris* | *Chlamydomonadaceae* | *Chlamydomonadales* | *Chlorophyceae* | *Chlorophyta* |
| 08610d18ebc3ca811af714e6b408f20b | 14.40 | 0.01 | 0.15 | 0.17 | *Chloromonas nivalis* | *Chlamydomonadaceae* | *Chlamydomonadales* | *Chlorophyceae* | *Chlorophyta* |
| 6ed5b6524bb2fa2aedbd829c72fa9eb9 | 3.33 | 2.33 | 0.00 | 1.11 | *Chlamydomonas nivalis* | *Chlamydomonadaceae* | *Chlamydomonadales* | *Chlorophyceae* | *Chlorophyta* |
| 2aa10d1c98c0729a9e862653855f8697 | 0.00 | 0.00 | 0.00 | 3.61 | *Malassezia globosa* | *Malasseziaceae* | *Malasseziales* | *Exobasidiomycetes* | *Basidiomycota* |
| eab90f2270b7b787b648c39c3f12966a | 6.50 | 0.05 | 0.05 | 1.88 | *Leucosporidium antarcticum* | *Leucosporidiaceae* | *Leucosporidiales* | *Microbotryomycetes* | *Basidiomycota* |
| b0e596639c7d1fc7131b11423593126c | 0.42 | 2.48 | 1.99 | 0.29 | *Rhodosporidiobolus fluvialis* | *Sporidiobolaceae* | *Sporidiobolales* | *Microbotryomycetes* | *Basidiomycota* |
| 90115a7ba5901e5cc3a3bd8f5e0df0d7 | 0.00 | 0.00 | 0.00 | 2.75 | *Peronospora belbahrii* | *Peronosporaceae* | *Peronosporales* | *Oomycetes* | *Oomycota* |
| 9b6eea2c450e59d3ac8d45a43cc300b1 | 0.21 | 2.09 | 0.40 | 0.72 | *Rhodotorula glutinis* | *Sporidiobolaceae* | *Sporidiobolales* | *Microbotryomycetes* | *Basidiomycota* |
| af55015aaf3ad1630fac766d8e43e13e | 0.00 | 0.02 | 7.40 | 0.28 | Fungal sp. | unclassified fungus | unclassified fungus | unclassified fungus | unclassified fungus |

**Table S2. Taxonomic assignment of highly abundant features within the 16S rRNA gene amplicon dataset.** The mean relative abundance [%] for the respective group of samples is shown, including closest taxonomic assignment when blasting sequences against the NCBI nucleotide collection database. Darker colors indicate higher abundances of respective feature.

| **Feature ID** | **Green snowfield** | **Red snowfield** | **Orange snowfield** | **Freshwater** | **Species** | **Family** | **Order** | **Class** | **Phylum** |
| --- | --- | --- | --- | --- | --- | --- | --- | --- | --- |
| 564adb46dc431643b5234c7dc867503c | 19.21 | 30.52 | 0.01 | <0.00 | *Solitalea koreensis* | *Sphingobacteriaceae* | *Sphingobacteriales* | *Sphingobacteria* | *Bacteroidetes* |
| 60082725460ec2576c15129df7526b68 | 6.12 | 12.41 | 0.00 | <0.00 | *Solitalea koreensis* | *Sphingobacteriaceae* | *Sphingobacteriales* | *Sphingobacteria* | *Bacteroidetes* |
| d6cbeeffd6a3ac4426b3eddbf834f6a7 | 0.59 | 13.84 | <0.00 | 0.00 | *Solitalea koreensis* | *Sphingobacteriaceae* | *Sphingobacteriales* | *Sphingobacteria* | *Bacteroidetes* |
| 2d233d8565b9e009f0b9a8168c1af830 | 15.45 | 4.11 | 5.65 | 0.02 | *Aquaspirillum arcticum* | *Neisseriaceae* | *Neisseriales* | *Betaproteobacteria* | *Proteobacteria* |
| bb329a9d1474ad8a2344dbb88114fc03 | 1.38 | 0.44 | 23.49 | 0.33 | *Massilia psychrophila* | *Oxalobacteraceae* | *Burkholderiales* | *Betaproteobacteria* | *Proteobacteria* |
| 861e0a55a13a33bb77e42809320cdd04 | 11.19 | 2.76 | 0.01 | <0.00 | *Hymenobacter lapidarius* | *Flavobacteriaceae* | *Flavobacteriales* | *Flavobacteria* | *Bacteroidetes* |
| 347cca0dd59c45fffa1320818250ce64 | 0.22 | 4.77 | 0.00 | 0.00 | *Solitalea koreensis* | *Sphingobacteriaceae* | *Sphingobacteriales* | *Sphingobacteria* | *Bacteroidetes* |
| 83f680fc6221facbcdaddc6788b83a1b | 11.48 | 0.12 | 0.18 | 27.93 | *Sphingomonas echinoides* | *Sphingomonadaceae* | *Sphingomonadales* | *Alphaproteobacteria* | *Proteobacteria* |
| 740f590850db6d15b55d1dc7e61c7dea | 8.68 | 1.05 | 0.00 | 0.00 | *Aquaspirillum arcticum* | *Neisseriaceae* | *Neisseriales* | *Betaproteobacteria* | *Proteobacteria* |
| a924253e03d8e32c319f7ace4c3a4acc | 1.28 | 3.22 | 0.22 | <0.00 | *Ferruginibacter paludis* | *Chitinophagaceae* | *Sphingobacteriales* | *Sphingobacteria* | *Bacteroidetes* |
| b7690f942b90ae2547fedeea49f94ff9 | 0.06 | 0.35 | 7.66 | 0.02 | *Glaciimonas alpina* | *Oxalobacteraceae* | *Burkholderiales* | *Betaproteobacteria* | *Proteobacteria* |
| 9df43f8481c6043696121494d679522e | 0.01 | 2.03 | 1.85 | 0.11 | *Massilia eurypsychrophila* | *Oxalobacteraceae* | *Burkholderiales* | *Betaproteobacteria* | *Proteobacteria* |
| fd797ac1c1c36a415f9daa21fa5ff79f | 0.00 | 0.13 | 7.24 | 0.10 | *Massilia psychrophila* | *Oxalobacteraceae* | *Burkholderiales* | *Betaproteobacteria* | *Proteobacteria* |
| 9daf1aa1f8c64060a6213a2d9eccb54d | 0.01 | 1.87 | 1.70 | 0.00 | *Parasediminibacterium paludis* | *Chitinophagaceae* | *Sphingobacteriales* | *Sphingobacteria* | *Bacteroidetes* |
| abbaa41465a3250f40e64f76fe2b6606 | <0.00 | 2.39 | 0.00 | 0.00 | *Solitalea canadensis* | *Sphingobacteriaceae* | *Sphingobacteriales* | *Sphingobacteria* | *Bacteroidetes* |
| 7b877ef3195b709308275f4a69d3fc41 | 0.20 | 1.85 | 0.96 | 0.00 | *Ferruginibacter paludis* | *Chitinophagaceae* | *Sphingobacteriales* | *Sphingobacteria* | *Bacteroidetes* |
| aed49d1bac57b960398e066fbc4b3c43 | 4.83 | 0.21 | 0.79 | 0.06 | *Polaromonas jejuensis* | *Comamonadaceae* | *Burkholderiales* | *Betaproteobacteria* | *Proteobacteria* |
| 2167a4bd43cfc7f7b8a5ee0dc0a6c1c4 | 0.52 | 1.76 | 0.13 | <0.00 | *Heliimonas saccharivorans* | *Chitinophagaceae* | *Sphingobacteriales* | *Sphingobacteria* | *Bacteroidetes* |
| d7fc725cfc10426d401ea2a9e3729671 | 5.53 | <0.00 | 0.00 | 7.75 | *Psychromicrobium silvestre* | *Micrococcaceae* | *Micrococcales* | *Actinobacteria* | *Actinobacteria* |
| 103530664a462938c5f77b52cc03e801 | 4.03 | <0.00 | 0.00 | 0.00 | *Hymenobacter lapidarius* | *Flavobacteriaceae* | *Flavobacteriales* | *Flavobacteria* | *Bacteroidetes* |

**Table S3. Statistical analyses of bacterial community composition in freshwater and snowfields sampled at two different locations based on the unweighted UniFrac distance matrix.**

| **ANOSIM test 16S** | | |
| --- | --- | --- |
| Groups | R | p-value |
| **Habitat**  **freshwater, snow** | **0.594** | **0.001** |
| **Snow color**  **red, green, orange** | **0.841** | **0.001** |
| Red snow  A- Rottenmann, B - Seetal | -0.015 | 0.501 |
| Freshwater  A- Rottenmann, B - Seetal | 0.194 | 0.116 |

**Table S4. Statistical analyses of eukaryotic community composition in freshwater and snowfields sampled at two different locations based on the unweighted UniFrac distance matrix.**

| **ANOSIM test 18S** | | |
| --- | --- | --- |
| Groups | R | p-value |
| **Habitat**  **freshwater, snow** | **0.223** | **0.008** |
| **Snow color**  **red, green, orange** | **0.903** | **0.001** |
| Red snow  A- Rottenmann, B - Seetal | 0.067 | 0.229 |
| Freshwater  A- Rottenmann, B - Seetal | 0.075 | 0.265 |

**Table S5. Detailed results of growth promotion experiments with the microalgae *C. vulgaris* and *C. typhlos* and bacterial strains isolated from snowfields and freshwaters.** Experiments were performed in 18-fold replication; results of co-cultivation experiments with *C. vulgaris* reaching statistical significance (*p*< 0.05) in growth-promotion are shown. Results of co-cultivation experiments with *C. typhlos* and the pre-selected bacterial strains are included. Increased algal biomass formation is given in percentage compared to axenic microalgae culture. ● Sampling site A (Rottenmanner Tauern); ■ Sampling site B (Seetaler Alpen). Color represents habitat of bacterial isolates (red – red snowfield, orange – orange snowfield, blue – freshwater). n.s. – not significant; n.a. – not analyzed.

| **Isolate** | **Algal CFU/mL [× 10^6^]**  **after co-incubation** | | **Difference in algal cell count compared to axenic culture [%]** | | **Siderophore**  **production** | **Auxin production**  **[>5 µg/mL]** | **AHL production** |
| --- | --- | --- | --- | --- | --- | --- | --- |
|  | ***C. vulgaris*** | ***C. typhlos*** | ***C. vulgaris*** | ***C. typhlos*** |  |  |  |
| **Axenic control** | 2.75 ± 0.60 | 0.54 ± 0.09 | n.a. | n.a. | n.a. | n.a. | n.a. |
| ● *Pseudomonas* sp. 1Ab3 | 4.97 ± 0.44 | 0.07 ± 0.02 | + 81 ± 16 | - 87 ± 22 | + | + | - |
| ● *Aeromonas* *salmonicida* 2Bb9 | 5.36 ± 0.69 | 2.50 ± 0.60 | + 95 ± 25 | + 363 ± 24 | - | + | + |
| ● *Pseudomonas trivialis* 2Ca3 | 4.48 ± 0.71 | 1.94 ± 0.54 | + 63 ± 26 | + 259 ± 28 | + | + | + |
| ■ *Pseudomonas antarctica* 3Ab1 | 4.26 ± 0.52 | 2.36 ± 1.02 | + 55 ± 19 | + 338 ± 43 | + | + | - |
| ■ *Pseudomonas* sp. 3Ac8 | 5.71 ± 0.36 | 0.64 ± 0.28 | + 108 ± 13 | n.s. | + | + | - |
| ■ *Pseudomonas* sp. 3Ea4 | 6.48 ± 0.77 | 0.69 ± 0.39 | + 136 ± 28 | n.s. | + | - | - |
| ■ *Pseudomonas* sp. 3Eb1 | 4.81 ± 0.74 | 2.33 ± 0.63 | + 75 ± 27 | + 332 ± 36 | + | + | - |
| ■ *Pseudomonas veronii* 3Ba6 | 4.48 ± 0.69 | 1.15 ± 0.29 | + 63 ± 25 | + 113 ± 36 | - | + | - |
| ■ *Janthinobacterium* sp. 3Dc5 | 7.23 ± 0.66 | 0.59 ± 0.34 | + 163 ± 24 | n.s. | + | + | - |

**Table S6. The mean relative abundance of representative features of algae growth-promoting strains in the 16S amplicon dataset.** ● Sampling site A (Rottenmanner Tauern); ■ Sampling site B (Seetaler Alpen). Color represents habitat of bacterial isolates (red – red snowfield, orange – orange snowfield, blue – freshwater).

| **Isolate** | **Representative Feature ID** | **Mean rel. abundance [%]** | | | |
| --- | --- | --- | --- | --- | --- |
|  |  | **Red snow** | **Green snow** | **Orange snow** | **Fresh-water** |
| ● *Pseudomonas* sp. 1Ab3 | 51bdbc262c549bf4dd1bd63ead56862f | 0.57 | 0 | 0.18 | 0.01 |
| ● *Aeromonas* *salmonicida* 2Bb9 | e8a3eccd511ebdc315b734b16a3d4333 | 0 | <0.00 | 0 | <0.00 |
| ● *Pseudomonas trivialis* 2Ca3 | 51bdbc262c549bf4dd1bd63ead56862f | 0.57 | 0 | 0.18 | 0.01 |
| ■ *Pseudomonas antarctica* 3Ab1 | 51bdbc262c549bf4dd1bd63ead56862f | 0.57 | 0 | 0.18 | 0.01 |
| ■ *Pseudomonas* sp. 3Ac8 | 460abde6df34176fefbcf523245c58c9 | 0.03 | 0 | 0 | 0 |
| ■ *Pseudomonas* sp. 3Ea4 | 6428107321f281acb5d1dd6ddebcde54 | 0.01 | 0 | 0.02 | 0 |
| ■ *Pseudomonas* sp. 3Eb1 | 51bdbc262c549bf4dd1bd63ead56862f | 0.57 | 0 | 0.18 | 0.01 |
| ■ *Pseudomonas veronii* 3Ba6 | 66fe7b08dedb8be2f70da2d3bc1cf65d | 0 | <0.00 | 0 | <0.00 |
| ■ *Janthinobacterium* sp. 3Dc5 | 1ca898bc470ce685731f68ac29e95269 | 0.06 | 0 | 0.75 | 0.02 |

**
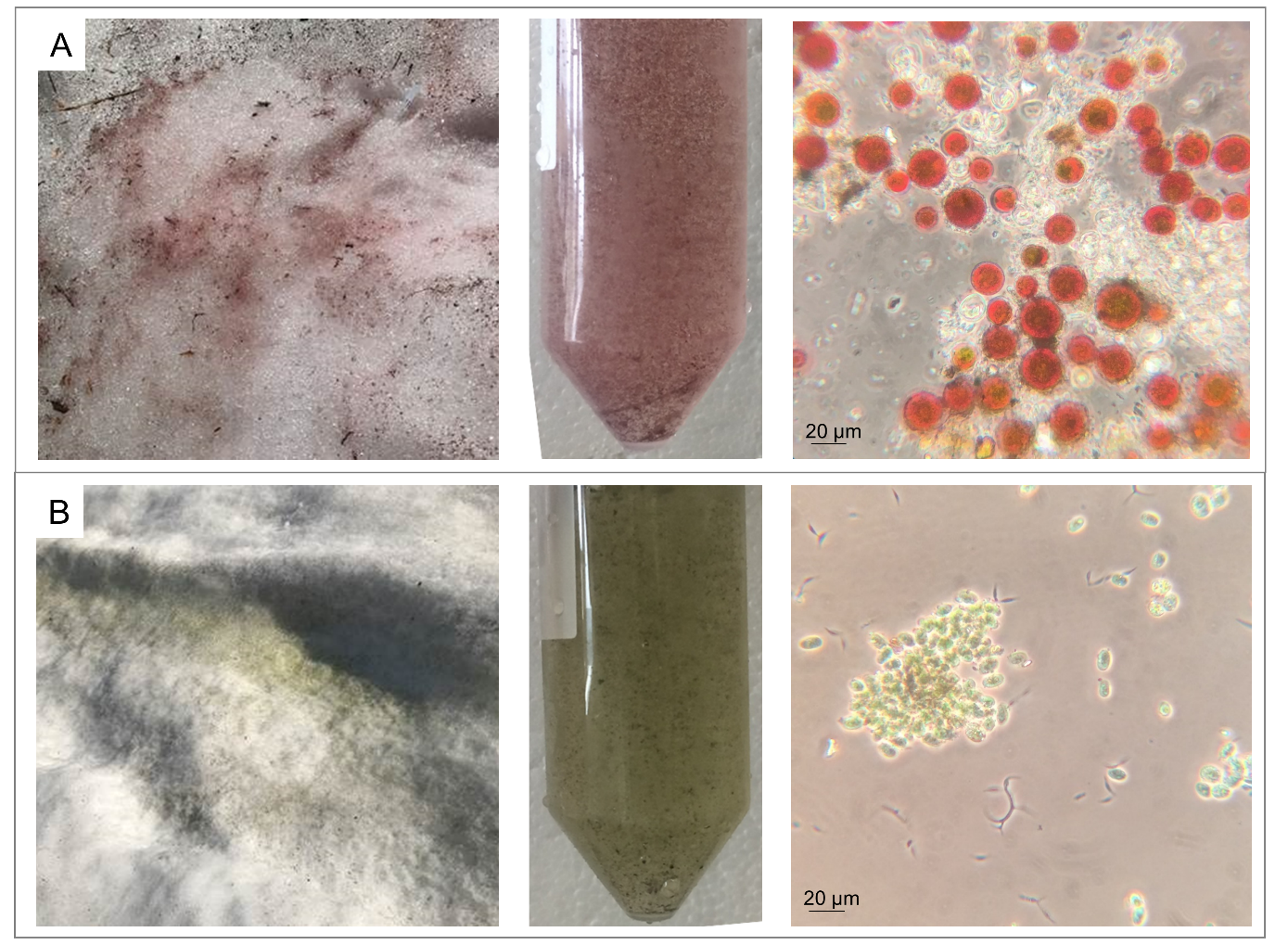
**

**Figure S1. Red (A) and green (B) snowfield sampled at site A (Rottenmanner Tauern).** Microscopic observation of melted snow gave first evidence of the presence of different microalgal species on differently colored snowfields.

**
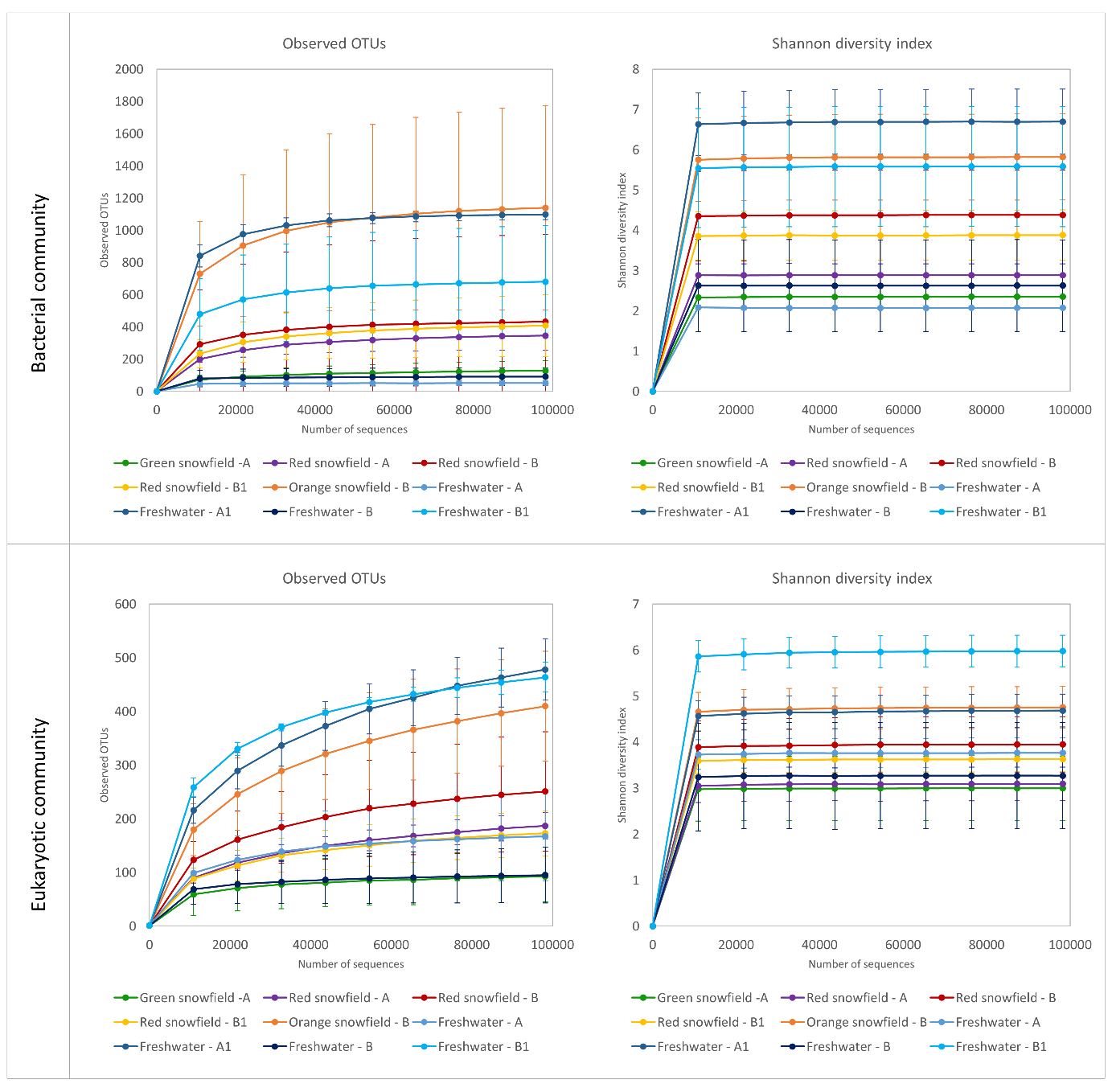
**

**Figure S2. The alpha diversity of the bacterial and eukaryotic community was assessed through observed OTUs and the Shannon diversity index.** Sampling site A - Rottenmanner Tauern; Sampling site B - Seetaler Alpen.

**
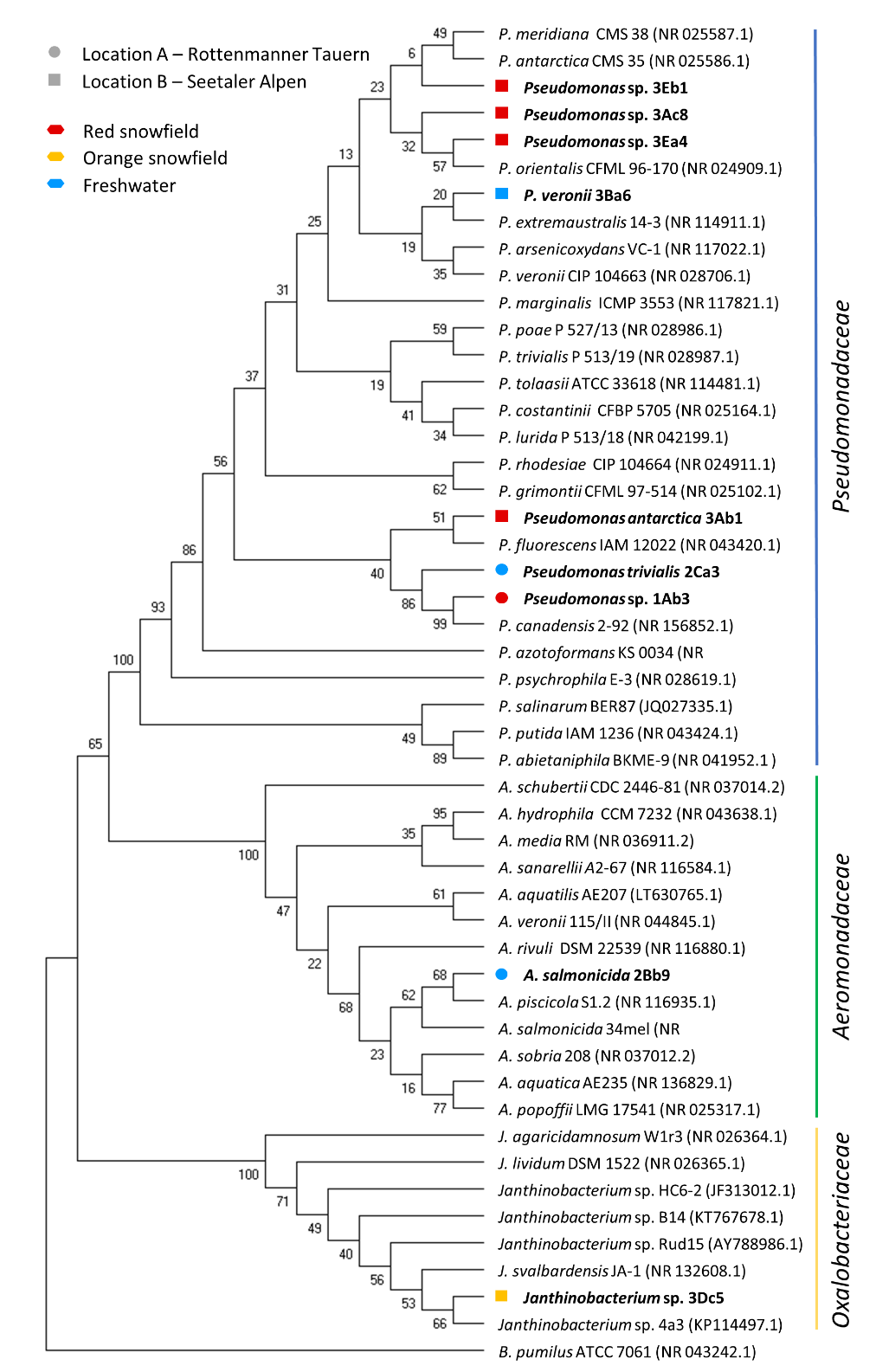
**

**Figure S3.** **Phylogenetic tree based on 16S rRNA gene fragment alignments, showing the positions of the isolates with microalgae growth-promoting properties among other isolates of *Pseudomonas*, *Aeromonas* and *Janthinobacterium***. The percentage of replicate trees in which the associated taxa clustered together in the bootstrap test (1000 replicates) are shown next to the branches. The analysis involved 50 nucleotide sequences. All ambiguous positions were removed for each sequence pair (pairwise deletion option). *B. pumilus* was used as outgroup. GenBank accession numbers are shown in parentheses.
