# Supplementary material for: The microbiome of alpine snow algae shows a specific inter-kingdom connectivity and algae-bacteria interactions with supportive capacities
Source: ISME J. 2020 May 18;14(9):2197–210. doi: 10.1038/s41396-020-0677-4 (PMC7608445; doi:10.1038/s41396-020-0677-4)
Supplement: Supplementary file 2 — Supplementary Material File 2 [file 41396_2020_677_MOESM2_ESM.docx]

**Supplementary Material File 2**

**The microbiome of alpine snow algae shows a specific inter-kingdom connectivity and algae-bacteria interactions with supportive capacities**

**Lisa Krug**^1^**^,2^, Armin Erlacher^1^, Katharina Markut^1^, Gabriele Berg^1^ and Tomislav Cernava^1*^**

*^1^Institute of Environmental Biotechnology, Graz University of Technology, Petersgasse 12, 8010 Graz, Austria*

*^2^ACIB GmbH, Petersgasse 14, 8010 Graz, Austria*

**Correspondence:*

*Tomislav Cernava, Institute of Environmental Biotechnology,*

*Graz University of Technology, Petersgasse 12,*

*8010 Graz, Austria.*

*e-mail:* [*tomislav.cernava@tugraz.at*](mailto:gabriele.berg@tugraz.at)

**Running title:** Interactions within alpine microbial communities

**Submitted to:** ISME Journal

**Subject Category:** Microbial ecology and functional diversity of natural habitats

**Keywords:** microalgae; microbiome; snowfield communities; freshwater microorganisms; microbial interplay

**Supplementary Material and Methods**

**Screening for N-acylhomoserine lactone (AHL) production.** Screening for AHL production was performed based on the studies of Morohoshi and colleagues (40) and McClean and colleagues (41). *Chromobacterium violaceum* CV026 served as indicator strain as it is able to produce the violet pigment violacein in the presence of exogenous N-hexanoyl-Lhomoserine lactone (C6-HSL). *S. plymuthica* 3Re4-18 (42) served as positive control. Isolates were grown over night at room temperature. Violet coloration of the indicator strain indicated AHL-production which was recorded for each strain.

**Screening for indole-3-acetic acid production.** Indole-3-acetic acid excretion by bacterial strains was determined by means of a modified colorimetric analysis developed by Gordon and Weber (43). Glass test tubes containing 5 ml LB broth supplemented with 0.1% tryptophan were inoculated with single colonies of bacterial isolates. After cultivation at 20 °C for 5 to 7 days in the dark, the cell-free supernatant was mixed with the Salkowski reagent (50.0 mM FeCl_3_, 35.0% (v/v) perchloric acid) at a ratio of 3:1 and incubated for 30 min in the dark. The auxin concentration was measured photospectrometrically using an infinite M200 spectrofluorimeter microplate reader (TECAN, Männerdorf, Switzerland) at 530 nm and quantified using a standard curve (R^2^ = 0.9894).

**Screening for siderophore production.** Siderophore production was tested by sterilizing 400 ml of 1.50% agar LB-agar medium and mixing it thoroughly with 100 ml staining solution containing 98 ml dH_2_O; 1 ml 0.01 M FeCl_3_ 1 ml 0.1 M HCl; 0.605 g chrome azurol-S and 0.073 g cetyltrimethylammoniumbromid. After solidification in Petri dish plates, bacterial isolates were streaked out and incubated for 14 days at room temperature. Then the presence (siderophore production positive) or absence (siderophore production negative) of yellow halo zones around the isolates was noted.

**Microalgal biomass quantification by fluorescence intensity analyses**

In order to establish a rapid quantification method, the algal chlorophyll A content was measured using a fluorometric approach and correlated with the respective cell number. The fluorescence emission maximum was determined by performing a florescence intensity scan with pure *C. vulgaris* and *C. typhlos* cultures using an infinite M200 spectrofluorimeter. The microalgae culture was obtained by inoculating 20 ml mBBM with a single colony of the respective microalgae and subsequent incubation for 5 days at 23 °C at a light dark cycle L:16/D:8. The microalgae culture was then excited at 450 nm wavelength and fluorescence emission was detected in the range between 580 and 760 nm. The fluorescence emission maximum of microalgae cultures was determined at 685 nm. The number of corresponding CFUs for each tested microalgae was determined by plating respective dilutions on mBBM. Applying a linear regression (*C. vulgaris,* R^2^ = 0.931; *C. typhlos* R^2^ = 0.908) allowed the correlation between fluorescence intensity (FI) and microalgae cell count.

**Supplementary Results**

**Eukaryotic communities show habitat-specific signatures in colored snowfields and freshwater samples**

The eukaryotic community structure was analyzed by 18S rRNA gene fragment amplicon sequencing of differently colored snowfields and freshwater samples from two geographically dispersed locations in the Austrian Alps (Fig. 2). After removing chimeric sequences, 10 374 680 reads were retained in the dataset, resulting in 3 681 features. Features were collapsed at genus level resulting in 71 eukaryotic genera with an occurrence of at least 10 reads in total. The resulting community assessment includes highly abundant eukaryotic taxa; their mean relative abundance within the sample replicates and a threshold of 0.1% over the whole dataset was used for visualizations (Fig. 3). The green and red snowfields sampled at sampling site A were dominated by *Archaeplastida* with relative abundances of 51% and 65% respectively, followed by *Ophistokonta* with relative abundances of 49% on the green snowfield and 33% on the red snowfield. A similar pattern was observed for the freshwater samples obtained at sampling site A; *Archaeplastida* accounted for 43% and *Ophistokonta* for 33% relative of the eukaryotic community, whereas *Basidiomycota* accounted for the largest share in freshwater. On red snowfields sampled at sampling site B *Ophistokonta* accounted for 82% followed by *Archaeplastida* with a relative abundance of 14% of the total eukaryotic community. The highest proportion of taxa assigned to *Ochrophyta* was found on the orange snowfield (7%). Within the phylum *Ochrophyta* not further classified *Ochromonadales* were frequently detected in one freshwater sample obtained at sampling site B with a relative abundance of 26%. *Bacillariophytina* (29%) and not further classified *Peronosporomycetes* (8%) - all members of the *Stramenopiles* clade - were the most abundant taxa in this sample. Similar results were obtained when analyzing the composition of the eukaryotic community of the second freshwater sample obtained at sampling site B. The most abundant eukaryotic taxa were members of the SAR clade (*Stramenopiles*, *Rhizaria*, *Alveolata*; 53%), whereas not further classified *Peronosporomycetes* was the dominating taxon (27%; Fig. 2). Detailed description of abundances for the most abundant features are shown in Table S1, Supplementary File; the mean relative abundance for the respective group of samples is displayed, including closest taxonomic assignment when BLAST searches were performed against the NCBI nucleotide collection database. When assessing the dominating algal genera on differently colored snowfields in more detail, red snowfields were clearly dominated by *Chlamydomonas*spp., while on green snowfields *Chloromonas* spp. prevailed; orange snowfields were predominantly inhabited by members of the *Chrysophyta* phylum. Results of cultivation-independent analyses were supported by microscopic observation of red and green snowfield samples (Fig. S1, Supplementary Material File); micrographs indicated the presences of *Chlamydomonas*spp. and *Chloromonas*spp. on red and green snowfields, respectively.

***Proteobacteria* and *Bacteroidetes* were predominant in bacterial communities on snowfields and in freshwater**

In a complementary approach based on the same total community DNA extracts, the bacterial community structure was analyzed by 16S rRNA gene fragment amplicon sequencing. After removing chimeric, plastid and mitochondrial sequences, 9 135 634 reads were retained in the dataset, resulting in 7 850 features. Features were collapsed on genus level and genera with an occurrence < 10 reads in the whole dataset were excluded, resulting in 539 bacterial genera. Bar charts were used to visualize the highly abundant fraction in the 16S dataset (relative abundance > 0.1%; Fig. 3). The resulting dataset comprised seven bacterial phyla that included 53 different bacterial genera. Among the red and green snowfield samples, the predominant fraction of the microbiota was assigned to *Bacteroidetes* (mean: 72%) followed by *Proteobacteria* (mean: 23%), together they accounted for ≥ 93% in each sample, independent from the sampling site. On genus level, the bacterial community on red and green snowfields was dominated by *Solitalea*sp. (29% - 80%). On the green snowfield a higher fraction of *Hymenobacter* sp. (15%) and *Aquaspirillum* sp. (24%) was observed compared to red snowfields. On the contrary, the orange snowfield sampled at sampling site B was dominated by *Proteobacteria* (66%) followed by *Bacteroidetes* (15%). The most abundant bacterial taxa on the orange snowfield were identified as members of the *Oxalobacteraceae* family (45%). Except one, freshwater samples were dominated by *Proteobacteria* (mean: 63%), in particular *Sphingomonas* (18% ‑ 80%), followed by *Bacteroidetes* (mean 14%). In one freshwater sample (Freshwater - B1) the dominating bacterial phyla were *Bacteroidetes* (46%) and *Proteobacteria* (39%; Fig. 3). The detailed description of relative abundances for the most abundant features is summarized in Table S2, Supplementary Material File; the closest taxonomic assignment according to BLAST searches against the NCBI nucleotide collection database was included.
